# CASE REPORT Simultaneous Latissimus Dorsi Myocutaneous Flap Transfer and Revision Total Knee Arthroplasty With Allograft Extensor Mechanism Reconstruction: A Case Report

**Published:** 2012-08-31

**Authors:** Daniel E. Verbist, Travis G. Boyd, Arthur L. Malkani, Bradon J. Wilhelmi

**Affiliations:** ^a^School of Medicine; ^b^Department of Orthopaedic Surgery; ^c^Division of Plastic and Reconstructive Surgery, University of Louisville, Ky

## Abstract

**Introduction:** We present the case of a patient undergoing simultaneous reconstruction of a massive soft tissue deficit of the right knee along with total knee arthroplasty and allograft reconstruction of the extensor mechanism after multiple failed attempts to repair and revise the affected joint. **Methods:** A latissimus dorsi myocutaneous flap was transferred to fill the soft-tissue deficit of the right knee. During the same procedure, a previously placed antibiotic-cement spacer was removed and a new total knee prosthesis was implanted. What remained of the damaged extensor mechanism was excised and replaced with a cadaveric allograft. **Results:** The latissimus dorsi flap provided the necessary soft-tissue coverage of the revision. The new knee components and allograft extensor mechanism were satisfactorily implanted. One year after simultaneous reconstruction, the knee remains functional and free of infection. **Discussion:** Although current literature may have indicated conversion to arthrodesis or prophylactic soft-tissue repair prior to revision, simultaneous soft-tissue and extensor mechanism repair along with revision total knee arthroplasty have yielded promising results in this patient.

Total knee arthroplasty (TKA) is one of the most commonly performed orthopedic procedures. More than 170,000 total knee arthroplasties were performed in the United States in 2001. Within the first 6 months after surgery, more than 5% of these joints developed one or more postoperative complications.^1^ Common postoperative complications of total knee arthroplasty include progression of underlying joint disease, trauma to the joint, loosening of the implant device, and implant infection.^2^ In many cases of complication, a revision TKA may be necessary, with infection being the most common indication.^3^ In other cases, however, revision may not be an option. Overwhelming soft-tissue deficits secondary to postoperative infection or destruction of the extensor mechanism may preclude the possibility of revision.^4^ The decision must then be made to attempt reconstruction of the soft-tissue and/or extensor mechanism or to convert the failed TKA to arthrodesis. Several options are available to the plastic surgeon if soft-tissue reconstruction is desired, although viability of donor sites and timing of the procedure may limit these options.

## METHODS

This patient is a 45-year-old man with a history of multiple knee joint operations including the removal of an infected knee prosthesis and placement of an antibiotic cement spacer. He suffered significant soft tissue loss in the region of the joint secondary to repeat surgical procedures, including most of the extensor mechanism, and demonstrated delayed wound healing after spacer placement. Following approximately 6 weeks with the spacer in place, the patient was once again taken to the operating room for revision total knee arthroplasty, free flap transfer, and extensor mechanism repair.

After removal of the cement spacer, a new total knee prosthesis was implanted, and the extensor mechanism was reconstructed with a patellar tendon allograft. At the time of the procedure, the patient had thin, contracted skin and deficient soft tissue over the knee that provided inadequate coverage of the prosthesis and allograft, and thus it required simultaneous reconstruction of the joint capsule.

A latissimus dorsi musculocutaneous free flap was transferred to develop a new soft tissue joint capsule. The flap was repaired to the above-knee popliteal vessels, and a skin paddle was included for postoperative surveillance of flap viability. The flap was skin-grafted and dressed to conclude the operation.

## RESULTS

The new knee components and allograft extensor mechanism were satisfactorily implanted. The latissimus dorsi flap provided the necessary soft-tissue coverage of the revision. The knee was immobilized for 6 weeks postoperatively in a knee immobilizer to allow the flap circulation time to inosculate the allograft. After 6 weeks of immobilization, standard postprosthesis therapy was initiated and the patient regained knee motion of 0 to 30 degrees.

More than 1 year after simultaneous reconstruction, the knee remains functional and free of infection.

## DISCUSSION

In many cases of soft-tissue deficiency, bone loss, and extensor mechanism compromise resulting from TKA and multiple revisions, the required course of action is to convert to arthrodesis or proceed with a transfemoral amputation.^4^^-^6 If the decision is made to attempt further revision to preserve the knee joint, then the role of the plastic surgeon is to reconstruct adequate soft-tissue mass to envelope the prosthesis and to restore a functional extensor mechanism.

Various techniques have been described for restoring sufficient soft-tissue coverage for TKA. The technique utilized for any given case is largely determined by magnitude of soft-tissue insufficiency.^7^ If a myocutaneous flap is required, the use of a medial gastrocnemius rotational flap has been well-established as the first choice of treatment.^8^^-^10 A free flap is rarely indicated but may be necessary if no adequate donor sites are available within close proximity to the defect.^11^ Limited literature documenting the use of a free myocutaneous latissimus dorsi flap suggests a high rate of complication and variable success at achieving primary wound healing.^7^

Regardless of the method of reconstruction, a second major factor to be considered is the time allotted between soft-tissue repair and TKA. In high-risk patients, particularly those who have experienced postoperative complications from previous TKA, the most common course of action is perform a prophylactic soft-tissue reconstruction several months before attempting a revision TKA.^12^^,^^13^ This approach is not always a viable option, however, particularly if the patient is adamantly opposed to undergoing multiple procedures, in which case a simultaneous reconstruction and repair might be considered.

Reconstruction of a damaged or missing extensor mechanism must also be performed if a revision TKA is desired. Various techniques have been described for this endeavor, including utilization of vascularized fascia lata and allograft implantation.^14^^,^^15^ Allograft reconstruction may be considered if the patient has no viable tissue donor sites available, but this method has demonstrated high likelihood of poor outcome.^15^^,^^16^

In this case, the patient had endured multiple unsuccessful previous attempts to repair the affected joint and strongly desired a single procedure for reconstruction. He elected to undergo simultaneous reconstruction of the soft-tissue deficit of his right knee with a latissimus dorsi myocutaneous free flap along with a revision total knee arthroplasty and allograft reconstruction of the extensor mechanism. This particular method of reconstruction was attempted because of the lack of viable tissue at more traditional donor sites for soft-tissue repair.

Although both combined flap reconstruction with revision TKA and allograft extensor mechanism repair have demonstrated variable results in the literature, the knee joint in this particular case remains functional and complication-free more than 1 year after the procedure.

## Figures and Tables

**Figure 1:**
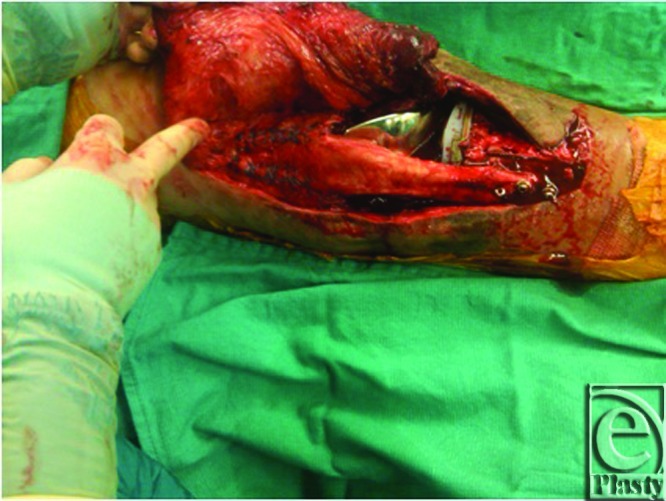
Implant arthroplasty and allograft patellar extensor unit.

**Figure 2:**
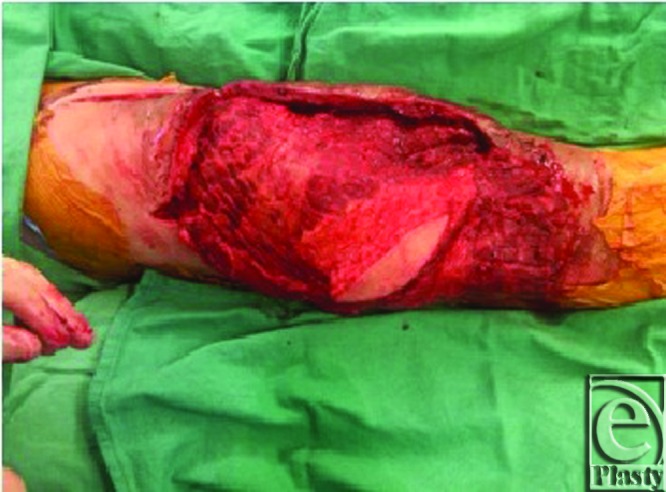
Latissimus dorsi musculocutaneous flap with skin paddle for flap monitoring.

**Figure 3:**
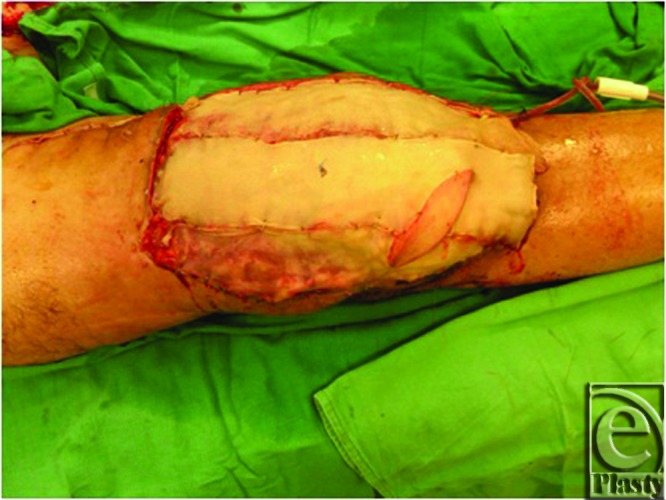
Split thickness skin grafting of latissimus dorsi flap.

**Figure 4:**
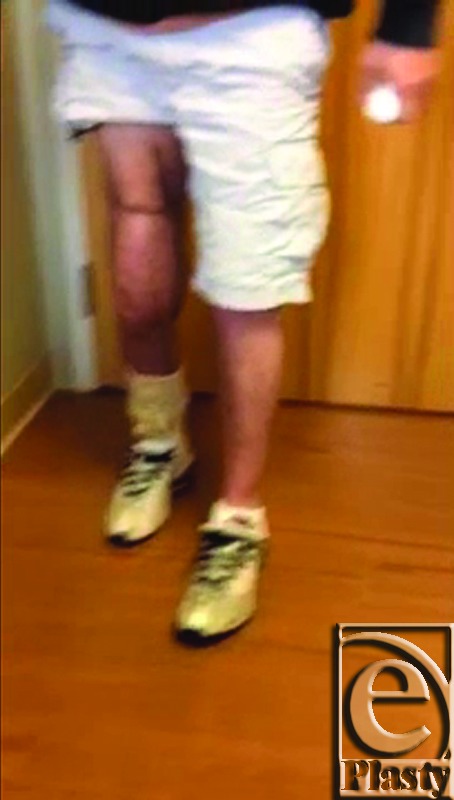
Patient walking on reconstructed right knee implant with muscle flap and allograft approximately 3 months after simultaneous reconstructive procedure. [Click Here to view video]
